# Adipocyte-derived stem cell-based gene therapy upon adipogenic differentiation on microcarriers attenuates type 1 diabetes in mice

**DOI:** 10.1186/s13287-019-1135-y

**Published:** 2019-01-22

**Authors:** Qing Fang, Min Zhai, Shan Wu, Xiaogen Hu, Zhan Hua, Huizhuo Sun, Jing Guo, Wenjian Zhang, Zai Wang

**Affiliations:** 1 Institute of Clinical Medical Sciences, China-Japan Friendship Hospital, Beijing, 100029 People’s Republic of China; 2 College of Chemical and Pharmaceutical Engineering, Hebei University of Science and Technology, Shijiazhuang, 050018 People’s Republic of China; 3 Research Center for Translational Medicine, Cancer Stem Cell Institute, East Hospital, Tongji University School of Medicine, Shanghai, 200120 People’s Republic of China; 4 Department of Plastic Surgery, China-Japan Friendship Hospital, Beijing, 100029 People’s Republic of China; 5 Department of General Surgery, China-Japan Friendship Hospital, Beijing, 100029 People’s Republic of China; 6 Beijing University of Chinese Medicine, Beijing, 100029 People’s Republic of China; 7 The 2nd Department of Pulmonary Disease in TCM, The Key Unit of SATCM Pneumonopathy Chronic Cough and Dyspnea, Beijing Key Laboratory of Prevention and Treatment of Allergic Diseases with TCM (No. BZ0321), Center of Respiratory Medicine, China-Japan Friendship Hospital; National Clinical Research Center for Respiratory Diseases, Beijing, 100029 People’s Republic of China

**Keywords:** Adipocyte-derived stem cell, Gene therapy, Adipogenic differentiation, Microcarrier, Diabetes

## Abstract

**Background:**

Insulin replenishment is critical for patients with type 1 diabetes; however, current treatments such as pancreatic islet transplantation and insulin injection are not ideal. In addition to stem cell or gene therapy alone, stem cell combined with gene therapy may provide a new route for insulin replenishment, which could avoid an autoimmune reaction against differentiated β cells or systematic viral vector injection.

**Methods:**

In this study, human adipocyte-derived stem cells (ADSCs) were transducted with lentiviral vectors expressing a furin-cleavable insulin gene. The expression levels of insulin were measured before and after adipogenic differentiation in the presence or absence of an adipocyte-specific promoter AP2. In vitro proliferation and in vivo survival of cells were examined on cytodex and cytopore microcarriers. The effect of ADSC-based gene therapy upon adipogenic differentiation on microcarriers was evaluated in the streptozotocin-induced type 1 diabetic mouse model.

**Results:**

We found that differentiation of ADSCs into adipocytes increased insulin expression under the EF1 promoter, while adipocyte-specific AP2 promoter further increased insulin expression upon differentiation. The microcarriers supported cell attachment and proliferation during in vitro culture and facilitate cell survival after transplantation. Functional cells on the cytopore 1 microcarrier formed tissue-like structures and alleviated hyperglycemia in the type 1 diabetic mice after subcutaneous injection.

**Conclusions:**

Our results indicated that differentiation of ADSC and tissue-specific promotors may enhance the expression of therapeutic genes. The use of microcarriers may facilitate cell survival after transplantation and hold potential for long-term cell therapy.

## Background

Insulin replenishment is the most common treatment for type 1 diabetes (T1D). However, T1D patients require regular and intensive insulin injection or must permanently carry an insulin pump. In addition, some patients have an increased risk of hypoglycemia [1]. The use of an artificial pancreas with continuous blood glucose monitors and a bi-hormone system reduces the risk of hypoglycemia; however, new safety concerns related to network or users may emerge [2]. Pancreas [3, 4] or islet transplantation [5, 6] can establish normal glycemia and avoid the occurrence of hypoglycemia. However, a shortage of donor organs and usage of immunosuppressive drugs greatly hamper the widespread implementation of pancreas or islet transplantation. Stem cells have a self-renewal capacity and can be differentiated into multiple cell types and, thus, hold potential for pancreatic β cell replacement therapy. However, low differentiation efficiency and immature and inhomogeneous status of the harvested cells, as well as immunoreactive problems and oncogenicity, impede the application of this method [7]. Another potential approach is to transduct target cells in vivo with insulin-expressing viral vectors in order to restore insulin production in other cell types other than pancreatic β cells [8–11]. In addition to stem cell or gene therapy alone, stem cell combined with gene therapy may provide a new route for insulin replenishment, which could avoid an autoimmune reaction against differentiated β cells or systematic viral vector injection [12].

Adipocyte-derived stem cells (ADSCs) can be easily obtained from autologous liposuction. These cells possess the major characteristics of mesenchymal stem cells (MSCs)—such as proliferation and differentiation ability—and have an immunoregulation function during inflammation [13]. Therefore, ADSCs have been considered as a good candidate vector for cell-based therapy. Research has shown that MSCs with gene modification could increase their therapeutic functions. For example, sustained secretion of exogenous IGF-1 may improve infarcted heart function [14]; expression of HGF could be a useful strategy for ischemic heart disease [15]; and transplantation of ADSCs expressing of VEGF165 may result in a more efficient angiogenic response in ischemic skeletal muscle [16]. Therefore, it is reasonable that MSCs, such as ADSCs expressing an insulin gene, could be a viable method of insulin replenishment. However, the expression level of this insulin gene in ADSCs and its derivative cell types remains to be examined.

Another issue in transplantation therapy is that to generate a long-term therapeutic effect, the vector cells should not be eliminated too quickly after transplantation. It has been reported that the number of ADSCs decreased significantly during the first week following fat pad transplantation and could hardly be detected after 28 days [17]. Besides the immune attack, anoikis (i.e., a programmed cell death process following detachment from the extracellular matrix) contributes to extensive cell death post-transplantation [18]. How to increase cell viability after transplantation remains a critical issue for stem cell-based insulin therapy.

Microcarriers are 125–250-nm beads that can support the growth of adherent cells in bioreactor suspension cultures. Their density is comparable to but slightly heavier than the culture medium, which allows them to be maintained in suspension with gentle stirring. The positive charge or matrix proteins on the surface support cell adherence and is thus beneficial to cell viability [19]. A commercial microcarrier cytodex has been used to attach to isolated islets and applied to a superfusion system, which avoided problems in a monolayer culture of islet cells [20]. Microcarriers have also been used in expansion cultures of MSCs in a bioreactor [21]. Interestingly, in vivo studies found that transplantation of adrenal chromaffin cells cultured on microcarriers improved cell growth and long-term function [22], implying that ADSC transplantation may also benefit from microcarrier culture.

In the current study, we compared the expression level of the insulin gene in ADSCs and their derivative adipocytes and increased insulin expression under an adipocyte-specific promoter in differentiated adipocytes. The pro-survival effect of commercial microcarriers was tested during the cell transplantation process, and the anti-diabetic effect was confirmed using the optimized approach in streptozotocin (STZ)-induced diabetic mice.

## Methods

### Isolation and culture of ADSCs

The study based on human ADSCs was approved by the Clinical Research Ethics Committee of China-Japan Friendship Hospital (No. 2015-45) and conducted according to the principles of the Declaration of Helsinki. ADSCs were collected by harvesting the fat tissue from liposuction or abdominal wall fat from laparotomy remnants. Following a wash in D-Hanks, the fat tissue was minced and digested in 0.1% collagenase I (Sigma Aldrich; Merck KGaA, Darmstadt, Germany) for 30 min at 37 °C with gentle agitation. Digestion was neutralized with complete cell culture medium. After centrifugation at 1200 rpm for 5 min, the cell pellet was resuspended in the culture media, filtered through a 100-μm cell strainer (BD Biosciences, Franklin Lakes, NJ, USA), and seeded in a T25 flask. The culture medium we used was DMEM/F12 (Hyclone, Logan, UT, USA) with 10% fetal bovine serum (Biological Industries Israel BeitHaemek, Kibbutz—BeitHaemek, Israel), 2.5 ng/ml bFGF (Peprotech, Rocky Hill, NJ, USA), 5 ng/ml EGF (Peprotech), and 1% penicillin-streptomycin (Hyclone).

### Flow cytometry assay of surface markers of ADSCs

Flow cytometry was performed following the constructions of Human MSC Analysis Kit (BD Biosciences). Briefly, the cells were trypsinized, neutralized with culture medium, washed with D-Hanks, and resuspended at a concentration of 1 × 10^7^/ml in D-Hanks containing 1% FBS. For each labeling, 100 μl of cell suspension was added in a FACS tube, and appropriate antibodies were added according to the protocol. Then, the tubes were incubated in the dark for 30 min on ice. After washes with centrifugation, the cells were analyzed on a flow cytometer (C6, BD Biosciences).

### Differentiation of ADSCs and histological analysis

For differentiation, ADSCs were grown to at least 90% confluence, and the medium was changed into differentiating medium. Adipogenic, osteogenic, and chondrogenic differentiation media were commercially available (Biowit Technologies, Shenzhen, China). For adipogenic differentiation, the cells were induced for 3–7 days with medium changed every 2–3 days. For detection of differentiated cells, the cells were fixed in 10% formalin for 10 min, stained with 0.5% (*w*/*v*) fresh Oil red-O solution (Sigma Aldrich) for 60 min at room temperature, and washed with D-Hanks for three times to detect the intracellular lipid droplets. For osteogenic differentiation assay, the cells were induced for 3 weeks, fixed in 10% formalin for 10 min, stained in 2% alizarin red (Sigma Aldrich) (pH 4.2) for 10 min, and washed with D-Hanks for three times to visualize the deposition of calcium minerals. For chondrogenic differentiation assay, the cells were induced for 2 weeks, fixed in 10% formalin for 10 min, stained in 1% Alcian Blue in 0.1 M HCl for 30 min at room temperature, and washed with 0.1% HCl for three times to visualized the acidic polysaccharides. The cells were monitored under an inverted light microscope (CKX41, Olympus, Tokyo, Japan).

### Expression vectors, lentivirus preparation, and infection

The lentiviral vector we used was derived from pcDH-EF1-MCS-T2A-puro (System Biosciences, Palo Alto, CA, USA). The T2A sequence was replaced with IRES sequence [23] amplified from pIRES2 (Clontech; Takara Bio USA, Inc., Mountain View, CA, USA) to avoid any addition of extra amino acids to the insulin coding sequence, and the resulted vector was termed as pcDH-EF1-MCS-IRES-puro. For EGFP labeling of the ADSC cells, EGFP gene was amplified from pEGFP-C1 (Clontech) vector and cloned into the MCS of the pcDH-EF1-MCS-IRES-puro.

To generate furin-cleavable human insulin, the amino acid sequence of insulin was first modified as follows. The cleavage site for PC1 at the B chain and C chain junction, KTRR, was changed to furin recognition site RTKR, and the cleavage site for PC2 at the C chain and A chain junction, LQKR, was changed to RQKR. B10D was also introduced to enhance the mature insulin stability [24]. The modified insulin sequence was codon optimized, synthesized, and cloned into pcDH-EF1-MCS-IRES-puro to generate pcDH-EF1-INS-IRES-puro. In some experiments, puromycin gene was replaced with EGFP gene to generate pcDH-EF1-INS-IRES-EGFP. To drive the adipose-specific expression of insulin, the essential promoter containing a 168-bp fragment upstream of the transcription starting site and the enhancer sequence from − 5.4 to − 4.9 kb of AP2 gene was synthesized according to the literature [25] and replaced the EF1 promoter (GenScript, Nanjing, China) to generate pcDH-AP2-INS-IRES-puro and pcDH-AP2-INS-IRES-EGFP.

For lentivirus generation, 293T cells were seeded 24 h before transfection, which would reach 80% confluence in 10-cm dish on the day of transfection. Transfection was performed using X-tremeGENE HP (Roche AG, Basel, Switzerland). Briefly, 10 μg expression vector, 4.5 μg pLP1, 3.5 μg pLP2, and 2.5 μg pVSVG were added into 1 ml opti-MEM (Gibco; Thermo Fisher Scientific, Inc., Waltham, MA, USA). Then, 60 μl transfection regent was added into the mixture and was mixed well. Twenty minutes later, the transfection mixture was added into the culture medium of 293T cells. Twelve to 18 hours later, the medium was changed into fresh medium. The supernatant was collected 48 h post-transfection, passed through a 0.45-μm filter, and ultracentrifuged at 90,000*g* for 90 min (XPN-80, Beckman Coulter, Brea, CA, USA). The viral pellet was resuspended in DMEM/F12 plus 10% FBS overnight and then applied to ADSC cells with 8 μg/ml polybrene (Sigma Aldrich). The infected cells were selected with 2 μg/ml puromycin 72 h later, or at this time point, green fluorescence was monitored under an inverted fluorescent microscope (BX51, Olympus).

### Microarray analysis

ADSCs differentiated towards adipocyte or undifferentiated were used for microarray analysis performed by CapitalBio Corporation (Beijing, China). GeneChip® PrimeView™ Human Gene Expression Array was used to detect the gene expression levels.

### Real-time RT PCR

Total RNA was extracted using RNA extraction kit (QIAGEN Inc., Valencia, CA, USA) according to the instructions. One microgram of total RNA was used for reverse transcription using FastQuant RT Kit with gDNase (Tiangen Biotech Co., Ltd., Beijing, China). Real-time PCR mixture was prepared using SYBR® Green Realtime PCR master mix (ToYoBo Co., Ltd., Osaka, Japan). The reaction was performed on an Applied Biosystems instrument (ABI 7500 system; Thermo Fisher Scientific, Inc.) for 40 cycles. Primers used are as follows: GAPDH forward: CTGCACCACCAACTGCTTAG, reverse: GAGCTTCCCGTTCAGCTCAG; AP2: forward: TGGGCCAGGAATTTGACGAA, reverse: GCGAACTTCAGTCCAGGTCA; and insulin forward: CTCACACCTGGTGGAAGCTC, reverse: AGAGGGAGCAGATGCTGGTA.

### Microcarrier-based culture of ADSCs

The microcarriers we used were cytodex 1, cytodex 3, and cytopore 1 (GE, Boston, MA, USA). The microcarrier was washed for three times with D-Hanks and stored in DMEM/F12 with 10% FBS. To generate microcarrier-based culture, an adequate amount of microcarrier was added into a non-adherent culture plate to cover the bottom of the plate. ADSCs were trypsinized and then added on to the microcarrier. This culture was established after incubation for 2 h to facilitate the cell attachment to the microcarrier with several times of mixing. To monitor the cell proliferation on the microcarriers, ADSC-EGFP cells were cultured on three types of microcarriers, and the fluorescent signals were measured by the fluorometer (SpectraMax Gemini XPS, Molecular Devices, San Jose, CA, USA). The empty microcarriers were used as background controls.

### Live image tracing of ADSC-derived cells in vivo

Eight-week-old male nude mice (nu/nu; Charles River, Beijing, China) were used in this experiment. Mice were maintained under SPF conditions and provided with food and tap water ad libitum. Mice were acclimatized to standardized laboratory conditions for about a week prior to experimentation (24 ± 2 °C; 50 ± 10% relative humidity; 12-h light-dark cycles). All animal studies were carried out in strict accordance with the Principles of Laboratory Animal Care and were approved by the Animal Studies Committee of the China-Japan Friendship Hospital (Beijing, China).

3 × 10^5^ cells in the 2D culture system or seeded on microcarriers were labeled with lipophilic tracer DiR [26] (Yeasen, Shanghai, China) for 20 min at 37 °C and washed with PBS for three times according to the instruction. The cells were injected into the nude mice. For cells without microcarriers, the cells resuspended in 100 μl DMEM/F12 were subcutaneously injected into the inguinal fat pad. For cells seeded on the microcarriers, they were resuspended in DMEM/F12, sucked into 2-ml syringe, and allowed to sink for a while. The extra medium was ejected, and the cells on microcarriers were injected as for the cells only. The mice were anesthetized with an intraperitoneal injection of 1% pentobarbital sodium (45 mg/kg) and then posed for near-infrared fluorescent live images (MIIS-XFP-STD, Molecular Devices).

### Cell therapy in T1D mouse model

T1D mice model was generated by intraperitoneal injection of 8-week-old male nu/nu mice with streptozotocin (STZ) (Sigma Aldrich) at 150 mg/kg in 0.1 M citrate buffer (pH 4.5) after an overnight fast. Blood glucose was monitored 1 week after STZ injection. Consecutive hyperglycemia with blood glucose > 16.7 mM was considered as diabetic. For the treatment of diabetic mice, 1 × 10^6^ cells on 200-μl microcarrier were injected into the mice as mentioned above.

### Quantification of insulin and C-peptide

To measure the secreted human C-peptide concentration in cell culture, the overnight culture supernatant was centrifuged to remove the cell debris and then evaluated by an EIA kit (DRG Instruments GmbH, Marburg, Germany). To measure the human insulin and C-peptide concentration in the mice plasma, blood was collected from the abdominal aorta of the mouse in an anesthetized condition at 28 days after treatment. The blood sample was centrifuged at 3000 rpm for 10 min to collect the serum. The concentrations of human insulin and C-peptide were measured by EIA kits accordingly (DRG Instruments GmbH).

### Statistical analysis

Values are expressed as means ± standard deviation (SD). Differences between the groups were examined by one-way ANOVA, followed by two-tailed Student’s *t* tests using SPSS® software (IBM, Armonk, NY, USA). *p* < 0.05 was considered significant.

## Results

### Characterization of adipose-derived stem cells

**Fig. 1:**
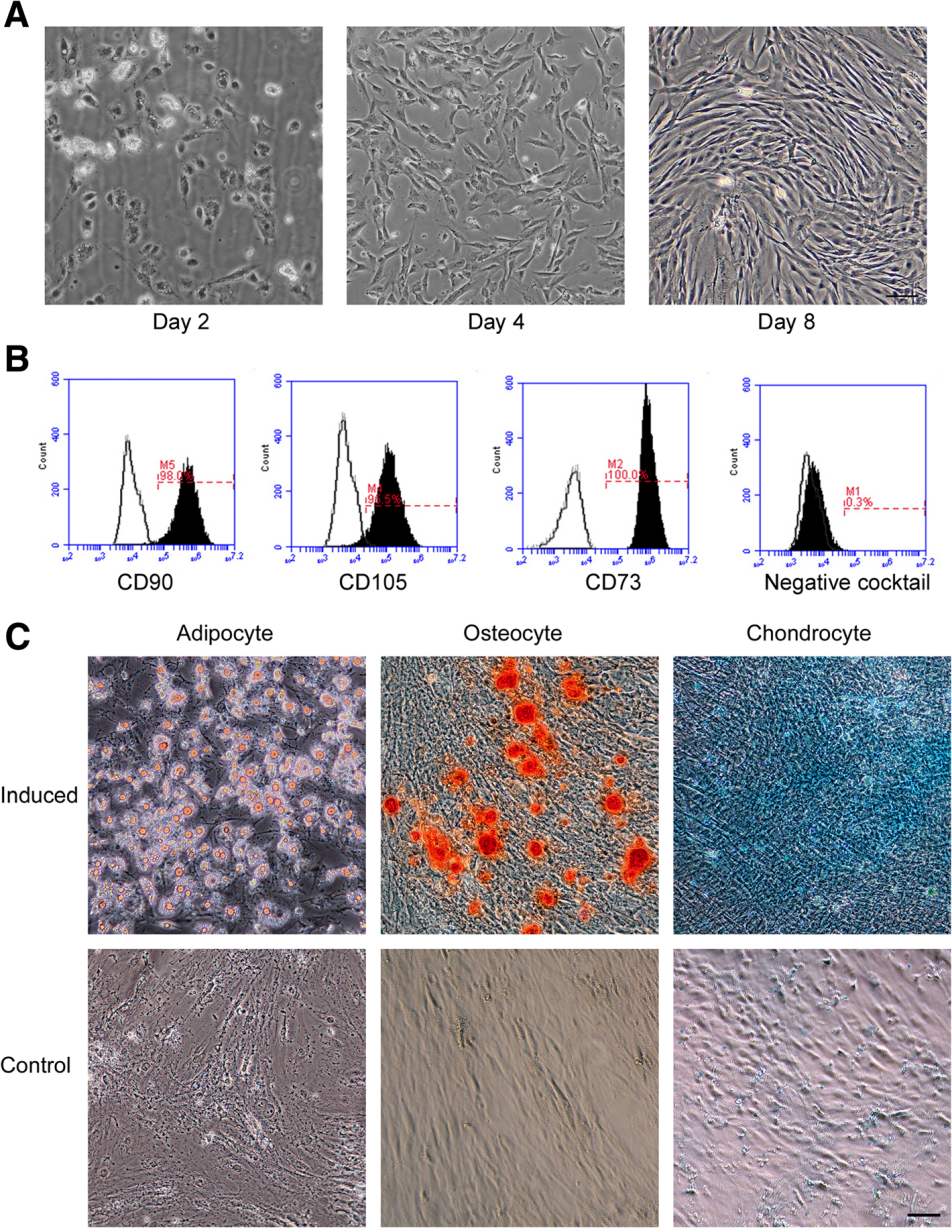
Characterization of adipose-derived stem cells (ADSCs). **a** Morphology of ADSCs. Cells isolated from fat tissues were cultured in vitro. Pictures were taken on days 2, 4, and 8 as indicated. **b** Flow cytometry analysis of mesenchymal stem cell markers. The positive markers including CD73, CD90, and CD105 and the negative markers including CD34, CD11b, CD19, CD45, and HLA-DR were analyzed. **c** Differentiation capacities of ADSCs. Cells were induced to differentiate towards adipogenic, osteogenic, and chondrogenic lineages, and Oil red-O, alizarin red, and Alcian Blue were used to detect the differentiation efficiency, respectively. Images were taken under an inverted light microscope. Undifferentiated cells were used as negative controls. Scale bar, 50 μm

Cells isolated from liposuction or surgery-derived fat tissues were cultured in vitro. Cells were round, polygonal, or fibroblast-like on day 2 after plating; elongated during the first week of culture; and appeared uniformly as fibroblast-like cells at day 8 onward (Fig. 1a). We used cells at passage 4 for stem cell marker analysis. The cells were positive for the mesenchymal stem cell surface markers CD73, CD90, and CD105 and negative for CD34, CD11b, CD19, CD45, and HLA-DR as revealed by FACS analysis (Fig. 1b). Furthermore, these cells had the capacity to differentiate into adipogenic, osteogenic, and chondrogenic lineages (Fig. 1c). All these data demonstrated that these cells had the characteristics of adipose-derived MSCs.

### Adipogenic differentiation enhanced exogenous insulin expression

Next, we transduced the ADSCs with human insulin-expressing lentiviral vectors, in which insulin expression was driven by the EF1 promoter (ADSC-EF1-INS). Insulin expression was monitored by real-time PCR, and insulin secretion was measured by ELISA on both packaging 293T cells and transduced ADSCs. Although the insulin expression and C-peptide secretion could be detected in ADSCs, they were at relatively low levels in comparison with 293T cells (data not shown).

**Fig. 2:**
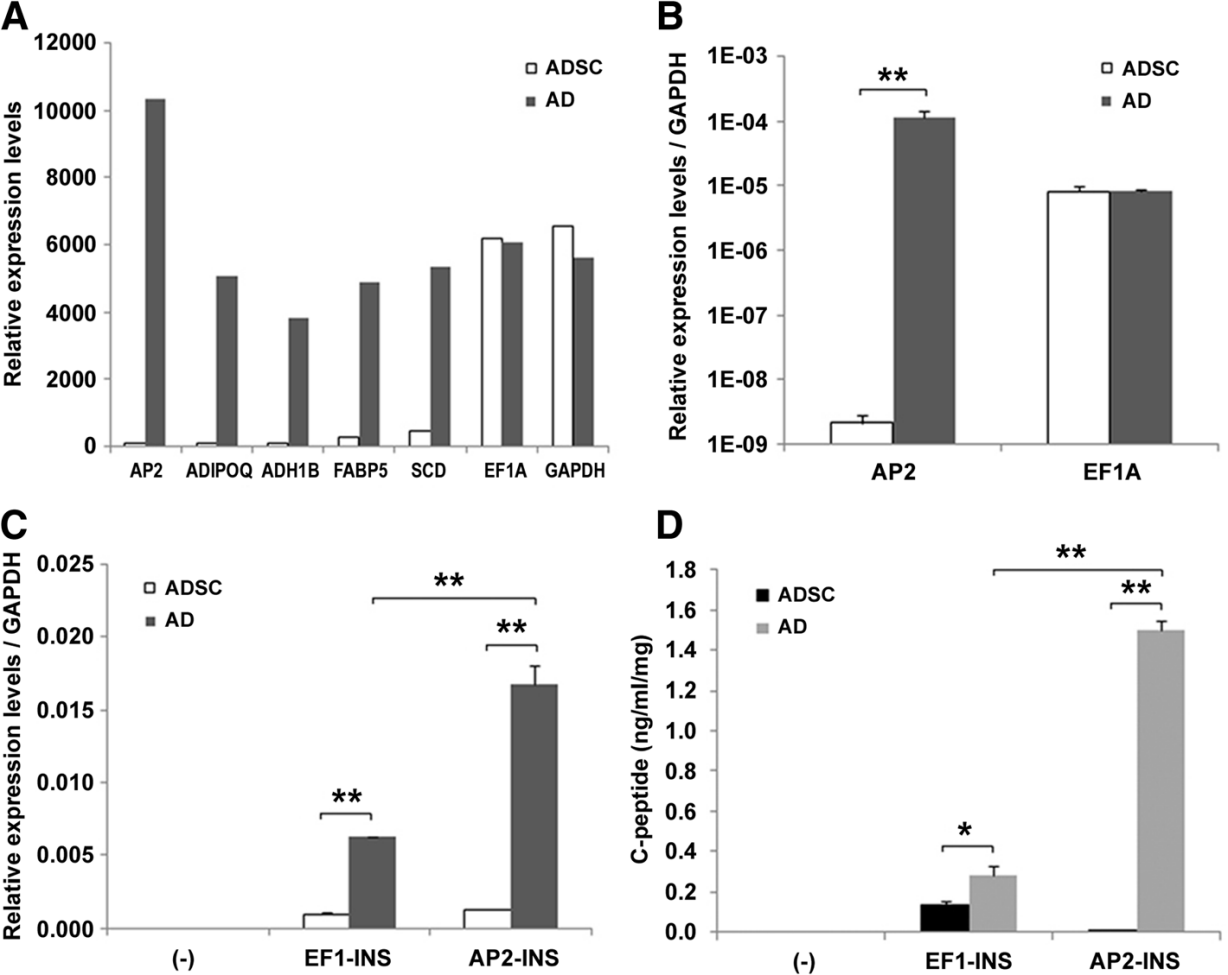
Effect of adipogenic differentiation on gene expressions. **a** Preliminary microarray analysis on ADSCs differentiated towards adipocyte (AD) or undifferentiated (ADSC). Selected gene expression levels are shown. **b** Real-time PCR analysis of the gene expression levels of AP2 and EF1A in ADSC and AD. GAPDH was used as an internal control. Insulin mRNA levels (**c**) and C-peptide secretion levels (**d**) in furin-cleavable human insulin stably expressed ADSC and AD cells driven by EF1 or AP2 promoter. Data derived from three independent experiments were presented as mean ± SD. *p < 0.05 and **p < 0.01 were considered significant

As adipogenic differentiation was very efficient in ADSCs and adipose-specific protein expression levels may increase dramatically following differentiation, we decided to take advantage of the adipose-specific promoters to drive exogenous insulin expression. We did microarray analysis to compare the differentially expressed genes before and after adipogenic differentiation. Our preliminary data showed that *FABP4* (AP2) was the most upregulated gene after differentiation (Fig. 2a). The markedly alleviated expression of AP2 was confirmed by real-time PCR analysis, while the house-keeping gene *EF1A* was not obviously changed (Fig. 2b). Therefore, we chose the AP2 promoter to drive insulin expression after differentiation and established AP2 promoter-driven INS-expressing ADSC cells (ADSC-AP2-INS) via a lentiviral strategy. We performed adipogenic differentiation of these cells and named the resulted cells AD-AP2-INS cells. Then, we analyzed insulin mRNA levels in both ADSC-EF1-INS and AD-AP2-INS cells. The insulin expression level increased by about eightfold in AD-AP2-INS compared to ADSC-EF1-INS cells (Fig. 2c). Insulin secretion as monitored by C-peptide secretion was increased by about tenfold (Fig. 2d). At the same time, adipogenic differentiation also improved EF1-driven insulin expression and secretion by about twofold in AD-EF1-INS cells (Fig. 2c, d). These results indicated that undifferentiated ADSCs may not be a potent gene expression vector; appropriate differentiation may enhance exogenic gene expression especially when a tissue-specific promoter is used.

### Microcarriers support ADSC proliferation and facilitate cell survival after transplantation

**Fig. 3:**
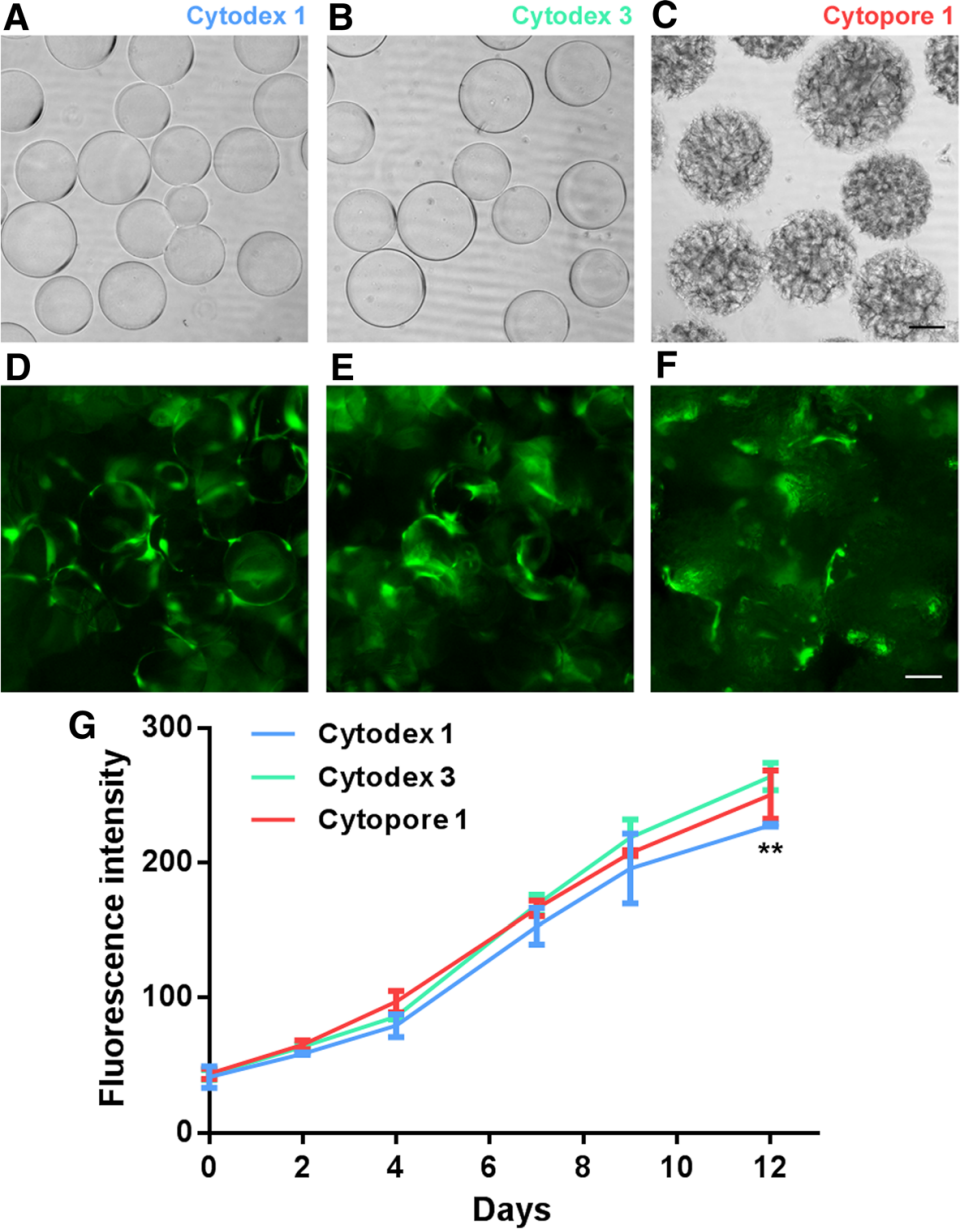
Microcarriers support ADSC growth. Morphology of microcarriers cytodex 1 (**a**), cytodex 3 (**b**), and cytopore 1 (**c**). Pictures were taken under bright field. ADSC-EGFP cells grown on cytodex 1 (**e**), cytodex 3 (**f**), and cytopore 1 (**g**). Pictures were taken under a fluorescent microscope. Scale bar, 50 μm. **g** The growth curve of ADSC-EGFP cells grown on microcarriers as indicated. The cell proliferation was monitored by the fluorescent signals. Data derived from three independent experiments were presented as mean ± SD. **p < 0.01, cytodex 1 vs cytodex 3, was considered significant

As documented, ADSCs or differentiated adipose cells soon diminished after transplantation on the fat pad, a big hurdle for long-term stem cell-based gene therapy. Except for rejection, which could be avoided when using autologous cells, the lack of cell attachment to the appropriate matrix may also accelerate cell death after transplantation. To solve this problem, we tested the ability of microcarriers (MCs)—including cytodex 1, cytodex 3, and cytopore 1—for ADSC culture and transplantation, which are regularly used in suspension cell cultures within bioreactors. Cytodex 1 and 3 are dextran-based solid spheres with average particle diameters of 180 and 175 μm, respectively (Fig. 3a, b). While cytodex 1 is positively charged, cytodex 3 is coated with collagen. Cytopore 1 is a cellulose-based macroporous sphere with an average particle diameter of 235 μm (Fig. 3c). We found that all three kinds of microcarriers can facilitate ADSC-EGFP attachment, survival (Fig. 3a–f), and proliferation as monitored by the EGFP fluorescent signals (Fig. 3g). For the two types of dextran beads, cytodex 3 supported faster cell growth than cytodex 1 (Fig. 3g) and, hence, was selected for further transplantation study.

**Fig. 4:**
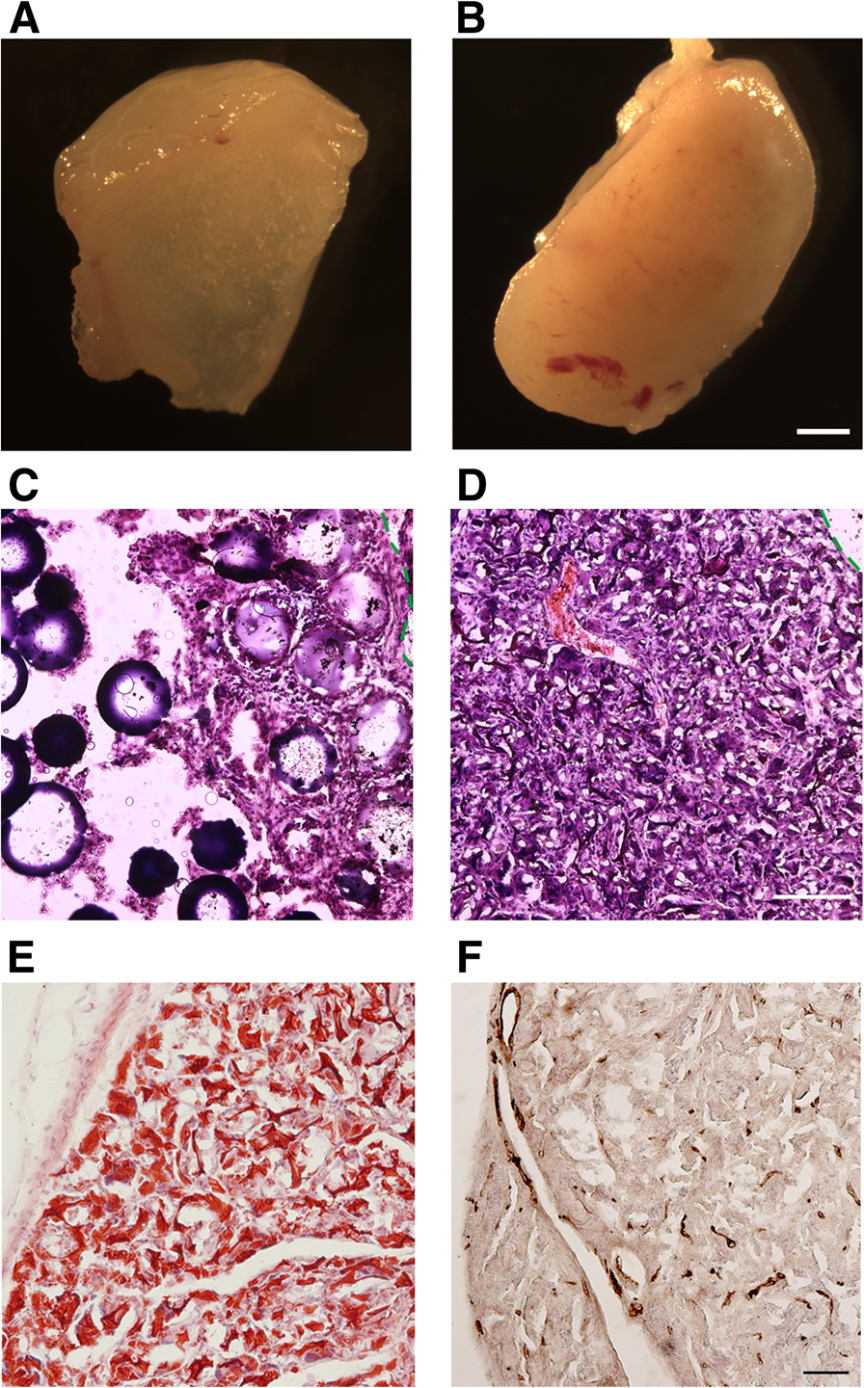
Tissue compatibility of cytodex 3 and cytopore 1. 0.2 ml empty microcarriers were subcutaneously injected into of the nude mice. Gross view of cross-sections of cytodex 3- (**a**) and cytopore 1 (**b**)-formed structures 2 weeks after injection. Scale bar, 1 mm. **c**, **d** H&E staining on frozen sections of cytodex 3- (**a**) and cytopore 1 (**b**)-formed structures. Green dashed lines show the border of the microcarrier-formed structures. Scale bar, 50 μm. **e**, **f** Compositional analysis of cytopore 1-formed structures. **e** The cellulose backbone of cytopore 1 was demonstrated by Congo red staining. **f** The endothelial cells were examined by CD31 immunohistochemistry. Scale bar, 50 μm

Next, we tested the tissue compatibility of cytodex 3 and cytopore 1 by subcutaneous injection of the empty microcarriers into nude mice. Two weeks after injection, the microcarrier-formed structures were removed and analyzed. Interestingly, while the solid cytodex 3 beads were loosely encapsulated by a fibrotic cyst without obvious blood vessel formation (Fig. 4a, c), the macroporous cytopore 1 beads formed a solid, tissue-like structure with abundant blood supply (Fig. 4b, d). The cellulose backbone of cytopore 1 could be detected by Congo red staining (Fig. 4e), and the endothelial cells of the invaded blood vessels could be detected by CD31 staining (Fig. 4f). These results demonstrated that the microporous structure of cytopore 1 permitted host cell penetration and tissue reconstitution. This formed the rationale for the selection of cytopore 1 as the microcarrier for subsequent experiments.

**Fig. 5:**
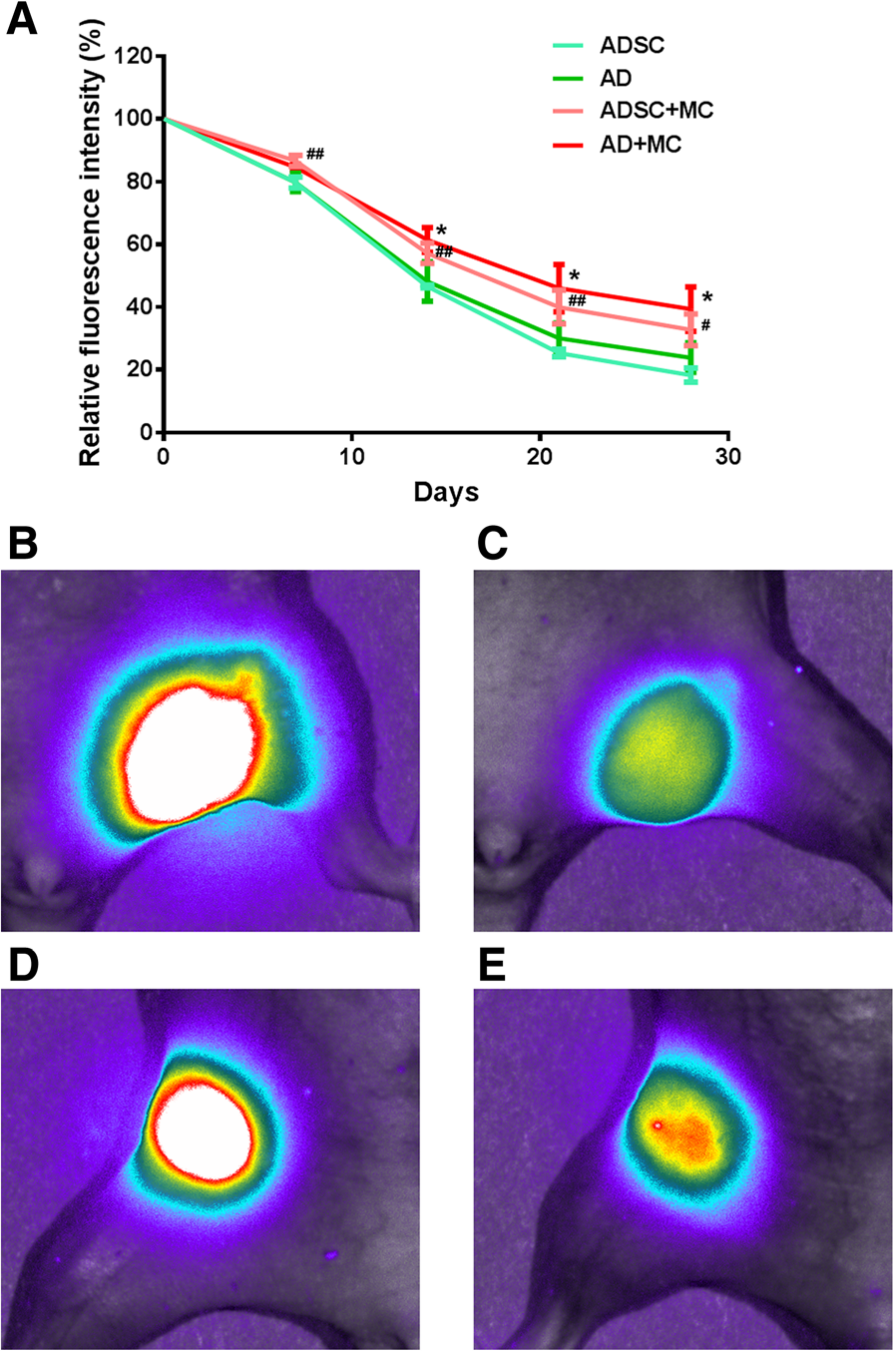
Comparison of the in vivo viability of ADSC-derived cells in the presence or absence of the cytopore 1 microcarrier. ADSCs or adipogenic-differentiated ADSCs (ADs) were labeled with DiR. Cells seeded on cytopore 1 microcarrier (MC) or not were subcutaneously injected into the inguinal fat pad of the nude mice (*n* = 3 for each group). **a** Cell viability calculated according to the fluorescent intensity at indicated time points. Data were presented as mean ± SD. *p < 0.05, AD+MC vs AD; ^#^*p* < 0.05; ^##^*p* < 0.01, ADSC+MC vs ADSC, was considered significant. Representative live image pictures of AD (**b**, **c**) and AD+MC (**d**, **e**) at day 0 (**b**, **d**) and 14 (**c**, **e**)

To compare the in vivo viability of ADSC-derived cells after transplantation in the presence or absence of the microcarrier, we labeled the cells with DiR, which gives near-infrared fluorescence allowing for live image tracing. The microcarriers were non-fluorescent (data not shown). We found that without a microcarrier, the fluorescent signals from DiR-labeled ADSCs or adipogenic-differentiated ADSCs (ADs) diminished to 18.23% and 23.83%, respectively; while in the presence of microcarriers, the fluorescent signals from ADSCs or ADs remained at 32.71% and 39.32% after 4 weeks, respectively (Fig. 5). The pre-inoculation of the cells on the microcarriers significantly increased cell viability after transplantation, both in differentiated and undifferentiated conditions, indicating that the cytopore 1 microcarrier facilitates the survival of ADSC-derived cells in vivo.

### AD-AP2-INS cells seeded on microcarriers alleviate hyperglycemia in STZ-induced type 1 diabetic mouse models

**Fig. 6:**
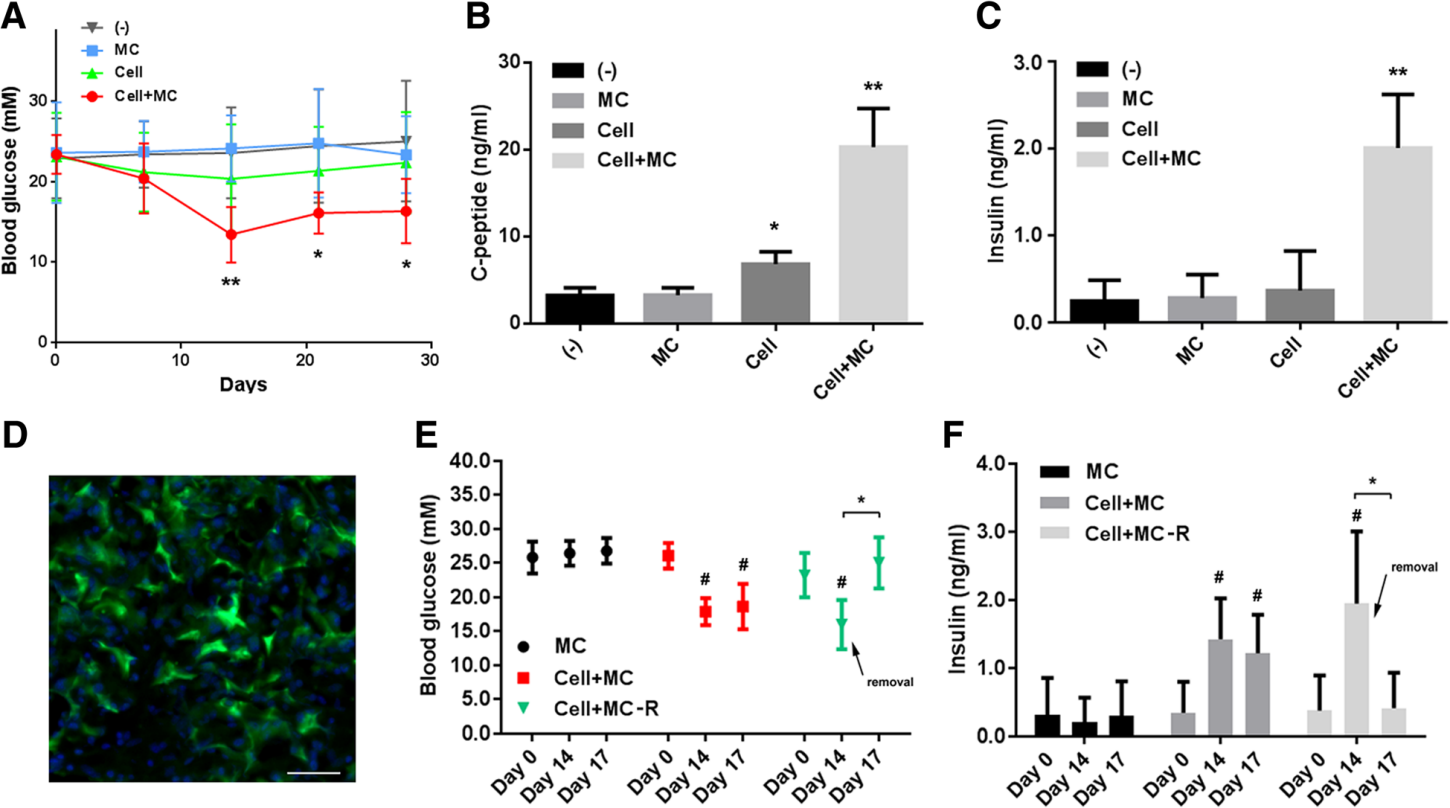
AD-AP2-INS cells seeded on microcarriers alleviate hyperglycemia in diabetic mouse models. **a**–**d** Empty cytopore 1 microcarrier (MC), AD-AP2-INS-EGFP cells (Cell), or AD-AP2-INS-EGFP cells seeded on MCs (Cell+MC) injected into the inguinal fat pad in STZ-induced diabetic mice (*n* = 5). **a** Blood glucose was monitored at indicated time points. C-peptide (**b**) and insulin (**c**) measured at 28 days after treatment. Data were presented as mean ± SD. *p < 0.05 and **p < 0.01, vs untreated control group (-), were considered significant. **d** Detection of transplanted cells on the microcarrier. Immunofluorescence against EGFP on the cross sections of a tissue-like structure formed in the Cell+MC group. Scale bar, 50 μm. **e**, **f** Empty cytopore 1 microcarrier (MC, *n* = 3) and AD-AP2-INS-EGFP cells seeded on MCs (Cell+MC and Cell+MC, *n* = 4) were injected into the inguinal fat pad in STZ-induced diabetic mice. In the group of MC+Cell-R, the MC+Cell was removed on day 14 as indicated by the arrows. Blood glucose (**e**) and insulin (**f**) were monitored at indicated time points. Data were presented as mean ± SD. ^#^*p* < 0.05 vs the MC group; *p < 0.05 as labeled was considered significant

Finally, we examined whether AD-AP2-INS cells on cytopore 1 microcarriers can alleviate type 1 diabetes in STZ-induced mouse models. We transplanted microcarriers (MC), AD-AP2-INS cells (Cell), or AD-AP2-INS cells seeded on MCs (Cell+MC) on the inguinal fat pad in STZ-induced diabetic mice by single injection. While the mice treated with microcarriers maintained hyperglycemia, the mice treated with AD-AP2-INS-EGFP cells seeded on microcarriers showed a significantly decreased blood glucose level from day 14 to day 28 after treatment (Fig. 6a). Mice treated with AD-AP2-INS cells showed a moderate anti-diabetic effect at day 14 after transplantation, but the effect could not last up to 28 days (Fig. 6a). C-peptide and insulin could be detected at significantly higher levels in the Cell+MC group versus the MC and Cell groups at day 28 after treatment (Fig. 6b, c). The transplanted AD-AP2-INS-EGFP cells could be traced by the EGFP marker protein in microcarrier-based structures (Fig. 6d), while no EGFP signal could be detected in the MC group (data not shown). Due to the small cell volume of AD-AP2-INS-EGFP cells lacking microcarriers, we did not detect any EGFP cells using section-based histological methods (data not shown). To confirm the function of the transplant further, we performed another set of experiments and removed the tissue-like structure formed in the Cell+MC group at day 14 after transplantation. The blood glucose level increased (Fig. 6e), and the insulin concentration decreased (Fig. 6f) to a comparable level to the MC group at day 17. Our results demonstrated that the transplanted functional cells on the microcarriers could contribute to long-term alleviation of diabetic hyperglycemia.

## Discussion

In this study, we combined stem cell and gene therapy strategies to treat type 1 diabetes in a mouse model. ADSC-derived adipose cells were used as insulin-expressing cell vectors, and the cytopore 1 microcarrier was used for cell attachment before transplantation. AD-AP2-INS cells on the microcarrier could alleviate hyperglycemia in the STZ-induced diabetic mice 2 weeks after a simple subcutaneous injection. The secretion of insulin and C-peptide could still be detected at 4 weeks after transplantation.

For cell-based insulin replenishment T1D treatment, several factors should be addressed. In our experiment, we chose gene therapy instead of stem cell differentiation for the insulin supply. It has been reported that embryonic stem cells [27], pancreatic progenitor cells [28], and MSCs [29] can be differentiated into pancreatic β cells or islet precursor cells. However, the differentiation efficiency and maturity of the resulting cells remained a challenge. In addition, differentiation into mature pancreatic β cells may lead to an increased risk of an autoimmune response and is not suitable for the treatment of an autoimmune disease such as T1D. Therefore, cell types other than pancreatic β cells were considered for ectopic insulin expression and release. In addition, along with proinsulin expression and processing, the C-peptide could be co-secreted with insulin, which may function in alleviating diabetic complications [30].

For the cell source for transplantation, bone marrow stem cells (BMSCs), ADSCs, and umbilical cord mesenchymal stem cells (UC-MSCs) are potential stem cell candidates. Although UC-MSCs are easily banked and have superior proliferation potential [31], their allogeneic origin may result in an immune response after transplantation. Ideally, autologous BMSCs or ADSCs could be used for transplantation; however, ADSCs are more accessible than BMSCs. The liposuction method for ADSC harvest is less invasive than bone marrow aspiration and brings minimal discomfort to patients. In addition, ADSCs possess the longest culture period and the highest proliferation capacity when compared to other types of MSCs [32]. Intrasplenic injection of ADSCs could decrease blood glucose levels and improve glucose tolerance in diabetic mice, probably due to the immunomodulatory ability of ADSCs [33]. In our study, ADSCs from liposuction or laparotomy remnants were used. Cells from these two sources have similar proliferation capacities and differentiation potentials.

For stable expression of the insulin gene, we chose lentiviral vectors instead of adeno-associated viral (AAV) vectors. Although the AAV does not integrate into the host genome and is considered safe for clinical use, the episomal form of the vector may result in decreased expression during long-term observation [34]. Currently, the extensive use of lentiviral vectors in cancer immunotherapy further supported their safety and efficacy [35], and hence, these were adopted in our experiments.

For the human insulin expression vector, since non-neuroendocrine cells lack the endoproteases PC1/3 and PC2, the processing of proinsulin cannot be accomplished normally. Hence, the recognition sites for PC1/3 and PC2 have been modified for the ubiquitously expressed protease furin [24]. However, the expression level of furin-cleavable proinsulin was less than that of its parent gene and varied among cell types [36]. Here, our data indicated that the expression level of furin-cleavable proinsulin in ADSCs was relatively low, suggesting that ADSCs may not be an ideal cell type for insulin gene expression.

ADSCs differentiate into adipocytes with high efficiency, and the expression level of the adipocyte-specific gene AP2 was substantially upregulated. We observed increased insulin gene expression after the adipogenic differentiation of ADSCs. For further improvement of insulin gene expression, we used the adipocyte-specific AP2 promoter to drive the expression, which achieved an eightfold enhancement compared with ADSCs expressing an EF1 promoter-driven insulin gene. In the future, other cell types and cell type-specific promoters may be tested for the improvement of insulin expression. For instance, the hepatocyte may be a better target cell type in the case of competent differentiation efficiency, considering their epithelial origin, vigorous protein synthesis and secretion, and closer relationship to the pancreatic lineage [8, 9, 37].

Another issue that should be considered is the regulated insulin secretion. In our system, insulin was constantly secreted and could not be regulated by glucose concentration (data not shown). Recently, minimally engineered HEK-293 cells have been rendered with glucose-inducible transcription of GLP-1 or insulin, shedding light on the regulated insulin replenishment therapy [38]. However, the regulation of protein secretion at the level of transcription might be slower than in the pancreatic β cells, in which insulin degranulation occurred quickly after stimulation.

For the mesenchymal cell culture system, microcarriers have been successfully used for growing anchorage-dependent cells, which enable an easy scale up of the cells for therapeutic applications [39]. In addition, anchorage-dependent cells undergo rapid cell death post-transplantation, probably due to anoikis [18]. Providing the cells with a substrate, such as the microcarrier, to allow attachment prior to transplantation could result in decreased anoikis and increased cell survival [40–43]. Here, we confirmed the capability of ADSC proliferation on three types of microcarriers and found that the microporous cytopore 1 formed a tissue-like structure with an abundant blood supply post-transplantation. Moreover, this tissue-like structure allowed easy tracking of the transplanted cells, as the structures are easily identified due to the adequate volume and are processible during subsequent histological analysis. More importantly, the microcarrier facilitated cell survival after transplantation.

## Conclusions

ADSCs are a good resource for combinatory cell and gene therapy. Differentiation of ADSCs and tissue-specific promotors may enhance the expression of therapeutic genes. The use of microcarriers could facilitate cell survival after transplantation. Functional insulin-producing cells pre-inoculated on microcarriers alleviated blood glucose in diabetic mice and may represent a promising approach for T1D treatment.

## Abbreviations

AD: Adipocyte
ADSC: Adipocyte-derived stem cell
MC: Microcarrier
MSC: Mesenchymal stem cell
SD: Standard deviation
STZ: Streptozotocin
T1D: Type 1 diabetes
